# Impact of time to intubation on mortality and pulmonary sequelae in critically ill patients with COVID-19: a prospective cohort study

**DOI:** 10.1186/s13054-021-03882-1

**Published:** 2022-01-10

**Authors:** Jessica González, Iván D. Benítez, David de Gonzalo-Calvo, Gerard Torres, Jordi de Batlle, Silvia Gómez, Anna Moncusí-Moix, Paola Carmona, Sally Santisteve, Aida Monge, Clara Gort-Paniello, María Zuil, Ramón Cabo-Gambín, Carlos Manzano Senra, José Javier Vengoechea Aragoncillo, Rafaela Vaca, Olga Minguez, María Aguilar, Ricard Ferrer, Adrián Ceccato, Laia Fernández, Ana Motos, Jordi Riera, Rosario Menéndez, Darío Garcia-Gasulla, Oscar Peñuelas, Gonzalo Labarca, Jesús Caballero, Carme Barberà, Antoni Torres, Ferran Barbé, CIBERESUCICOVID Project (COV20/00110, ISCIII), Ricard Ferrer, Adrián Cecato, Laia Fernández, Anna Motos, Jordi Riera, Rosario Menéndez, Dario García-Gasulla, Oscar Peñuelas, Gonzalo Labarca, Jesus Caballero, Carme Barberà

**Affiliations:** 1 Pulmonary Department, Hospital Universitari Arnau de Vilanova and Santa Maria, Av. Alcalde Rovira Roure, 80, 25198 Lleida, Spain; 2 Translational Research in Respiratory Medicine Group (TRRM), Lleida, Spain; 3 Lleida Biomedical Research Institute (IRBLleida), Lleida, Spain; 4 CIBER of Respiratory Diseases (CIBERES), Institute of Health Carlos III, Madrid, Spain; 5 Intensive Care Department, Vall d’Hebron Hospital Universitari, Barcelona, Spain; 6 SODIR Research Group, Vall d’Hebron Institut de Recerca (VHIR), Barcelona, Spain; 7 Pulmonary Department, Hospital Clinic, Universitat de Barcelona. IDIBAPS. ICREA, Barcelona, Spain; 8 University and Polytechnic Hospital La Fe, Valencia, Spain; 9 Barcelona Supercomputing Center (BSC), Barcelona, Spain; 10 Hospital Universitario de Getafe, Madrid, Spain; 11 Faculty of Medicine, University of Concepcion, Concepción, Chile; 12 Department of Clinical Biochemistry and Immunology, Faculty of Pharmacy, University of Concepcion, Concepción, Chile; 13 Intensive Care Department, Hospital Universitari Arnau de Vilanova de Lleida, IRBLleida, Lleida, Spain; 14 Intensive Care Department, Hospital Universitari Santa Maria de Lleida, Lleida, Spain

**Keywords:** COVID-19, ARDS, Critically ill patients, Early intubation, Respiratory management, Pulmonary sequelae

## Abstract

**Question:**

We evaluated whether the time between first respiratory support and intubation of patients receiving invasive mechanical ventilation (IMV) due to COVID-19 was associated with mortality or pulmonary sequelae.

**Materials and methods:**

Prospective cohort of critical COVID-19 patients on IMV. Patients were classified as early intubation if they were intubated within the first 48 h from the first respiratory support or delayed intubation if they were intubated later. Surviving patients were evaluated after hospital discharge.

**Results:**

We included 205 patients (140 with early IMV and 65 with delayed IMV). The median [p_25_;p_75_] age was 63 [56.0; 70.0] years, and 74.1% were male. The survival analysis showed a significant increase in the risk of mortality in the delayed group with an adjusted hazard ratio (HR) of 2.45 (95% CI 1.29–4.65). The continuous predictor time to IMV showed a nonlinear association with the risk of in-hospital mortality. A multivariate mortality model showed that delay of IMV was a factor associated with mortality (HR of 2.40; 95% CI 1.42–4.1). During follow-up, patients in the delayed group showed a worse DLCO (mean difference of − 10.77 (95% CI − 18.40 to − 3.15), with a greater number of affected lobes (+ 1.51 [95% CI 0.89–2.13]) and a greater TSS (+ 4.35 [95% CI 2.41–6.27]) in the chest CT scan.

**Conclusions:**

Among critically ill patients with COVID-19 who required IMV, the delay in intubation from the first respiratory support was associated with an increase in hospital mortality and worse pulmonary sequelae during follow-up.

**Supplementary Information:**

The online version contains supplementary material available at 10.1186/s13054-021-03882-1.

## Introduction

Severe acute respiratory syndrome coronavirus 2 (SARS-CoV-2) was identified in December 2019 as the cause of coronavirus disease 2019 (COVID-19) [1]. A far from negligible proportion of hospitalized patients (20–67%) may develop a more severe disease, resulting in acute respiratory distress syndrome (ARDS) [2, 3]. ARDS has generated a surge of patients who require respiratory support with invasive or noninvasive mechanical ventilation (IMV and NIMV) [3, 4]. The highest mortality rates are associated with IMV in patients with COVID-19, ranging from 16.7 to 88–97% [5]. Furthermore, respiratory impairment is very common in surviving critically ill patients with COVID-19 and well described [6–9]. After hospital discharge, the most frequent respiratory function abnormality (up to 82%) is an impairment in the carbon monoxide diffusing capacity (DLCO) [6]. Additionally, a higher proportion of patients (up to 70%) present a reticular or fibrotic pattern on chest CT scans at follow-up [6].

COVID-19-induced ARDS (CARDS) has been proposed as an “atypical ARDS” due to the dissociation of relatively well-preserved lung mechanics and the severity of hypoxemia [10, 11]. The management of CARDS has changed over time. At the beginning of the COVID-19 pandemic, most clinicians followed the recommendations of international guidelines for the treatment of CARDS using either standard oxygen therapy (SOT) or early IMV [12]. As the pandemic progressed, hospitals were overloaded and the number of ventilators was limited; thus, the trend to use noninvasive techniques such as NIMV or high-flow oxygen therapy by nasal cannula (HFNC) increased. Moreover, the strategy for using these techniques outside the ICU is even more widely accepted [13]. To date, the effectiveness and optimal respiratory support strategy for CARDS are still unknown.

The high mortality rate associated with CARDS appears to be decreasing [14, 15]; however, the inconsistent results have been emerged [16]. This discrepancy could be explained by many factors, but the decision on the management of respiratory support might play an important role. While some experts advocate for early intubation to combat patient self-inflicted lung injury (P-SILI) [10, 17–19], others defend exhausting noninvasive options before IMV [19–23]. Wendel Garcia et al. [24] recently published an important study conducted in the ICU showing that NIV was associated with higher mortality rates (HR: 2.67; 1.14–6.25; *p* < 0.001) than other respiratory support strategies.

Our study consists of a prospective cohort of ICU patients who needed to be intubated due to CARDS. With the aim of assessing the effect of early respiratory strategy, we compared in-hospital mortality and pulmonary sequelae in patients who were intubated within the first 48 h from the first ventilatory support (HFNC or NIV) and those intubated later (> 48 h). Pulmonary sequelae were evaluated at follow-up including pulmonary function tests (spirometry, lung volumes and DLCO), exercise tests (6MWT) and chest CT scans.

## Materials and methods

### Study design and population

This descriptive observational study was performed with all patients who had a critical care admission and orotracheal intubation (OI) due to COVID-19 at Hospital Universitari Arnau de Vilanova and Santa Maria in Lleida (Spain) between March 2020 and February 2021. The study is a subset of the ongoing multicenter study CIBERESUCICOVID (NCT04457505).

The study was approved by the Medical Ethics Committee (CEIC/2273) and complies with the tenets of the Declaration of Helsinki. Informed consent was acquired from the majority of patients using emergency consent mechanisms in accordance with the ethics approval guidelines for the study.

The main objective of this study was to determine whether the time of intubation from the first respiratory support affected (1) in-hospital mortality and (2) pulmonary sequelae during the follow-up of survivors.

### Inclusion and exclusion criteria

All patients were positive for SARS-CoV-2, were older than 18 years and had been admitted to the ICU and required OI at any time. Follow-up of patients who survived was based on the following exclusion criteria: (1) transfer to another institution during hospitalization, (2) treatment with palliative care, (3) follow-up in another department and (4) severe mental disability that made it impossible to assess pulmonary function.

### Clinical data collection

#### Baseline and ICU stay

Patient sociodemographic and comorbidity data were obtained. Clinical, vital, ventilatory and laboratory parameters were recorded at hospital and ICU admission. The latter include general blood tests for acute markers of inflammation.

The start dates of the first respiratory support with IMV, NIMV or HFNC were recorded whether it was provided in the general ward or in the ICU. ROX (Respiratory rate-OXygenation) index was calculated at the first respiratory support (NIMV or HFNC). Patients were divided into two groups: the early intubation group of patients who were intubated within the first 48 h from the first ventilatory support and the delayed group of those intubated later (> 48 h). We also collected data on the length of ICU and hospital stays, the duration of IMV and the need for and duration of prone positioning. APACHE score (Acute Physiology and Chronic Health Evaluation) was recorded at ICU admission. The institution protocol of the routine criteria for intubation was based on standard care and included:

- Hypoxemic respiratory failure with persistent need for high flows/fractions of inspired oxygen and evolving:
  o Hypercapnia, increasing work of breathing (RR > 30 rpm), increasing tidal volume, worsening mental status, increasing duration and depth of desaturations
- Hemodynamic instability or multiorgan failure.

Information of mechanical ventilation parameters such as tidal volume, end-inspiratory plateau and peak inspiratory pressures, positive end-expiratory pressure (PEEP), driving pressure and static compliance of the respiratory system (Crs) was recorded at the start of intubation.

#### Follow-up visit

Between the third and sixth months after hospital discharge, patients were evaluated by performing pulmonary function and exercise tests and a chest CT examination, as previously described [6].

### Statistical analysis

Descriptive statistics were calculated to describe sociodemographic and clinical characteristics, ICU-related information and post-COVID sequelae. Absolute and relative frequencies were calculated for qualitative variables, and medians (25th percentile; 75th percentile) were estimated for quantitative variables. Continuous variables and categorical variables were compared between groups using the Mann–Whitney U test and Fisher’s exact test, respectively.

To try to compensate the lack of randomization in this study a propensity score (PS) was performed. Propensity score was defined as the probability of belonging to the delayed group according to a logistic regression model. Age, sex and characteristics of the patients in the hospital that showed significant differences between groups were included in the model as predictors. All adjusted models used a doubly robust adjustment method including PS predictors as covariates. The odds ratio (95% CI) was estimated to compare in-hospital mortality between study groups. Adjusted ORs were estimated using a logistic regression model. For survival analyses, the time from the start of the IMV to the day of death and hospital discharge was used. A Cox model was used to estimate adjusted and unadjusted hazard ratios (HRs). The cutoff point was established to fit mortality risk using a maximally selected log-rank statistic [25]. An additive Cox proportional hazard model with time to IMV as continuous predictor was used to assess dose–response relationship with the mortality risk. We additionally performed a multivariate Cox model including important mortality predictive factors previously reported in the literature [24, 26] and adding delay in intubation. Additionally, we performed a competing risk analysis because in-hospital mortality risk can be overestimated when considering discharge as censored information [27, 28]. In-hospital mortality and discharge were evaluated with the competing risks analysis using the cumulative incidence function [29]. The proportional subdistribution hazard model using the Fine and Gray competing risk regression model was fitted to estimate the effect of covariates on in-hospital mortality. We explored in-hospital predictors of sequelae (measured using DLCO and TSS) by selecting factors that predicted in-hospital mortality based on a random forest model [30].

## Results

**Fig. 1:**
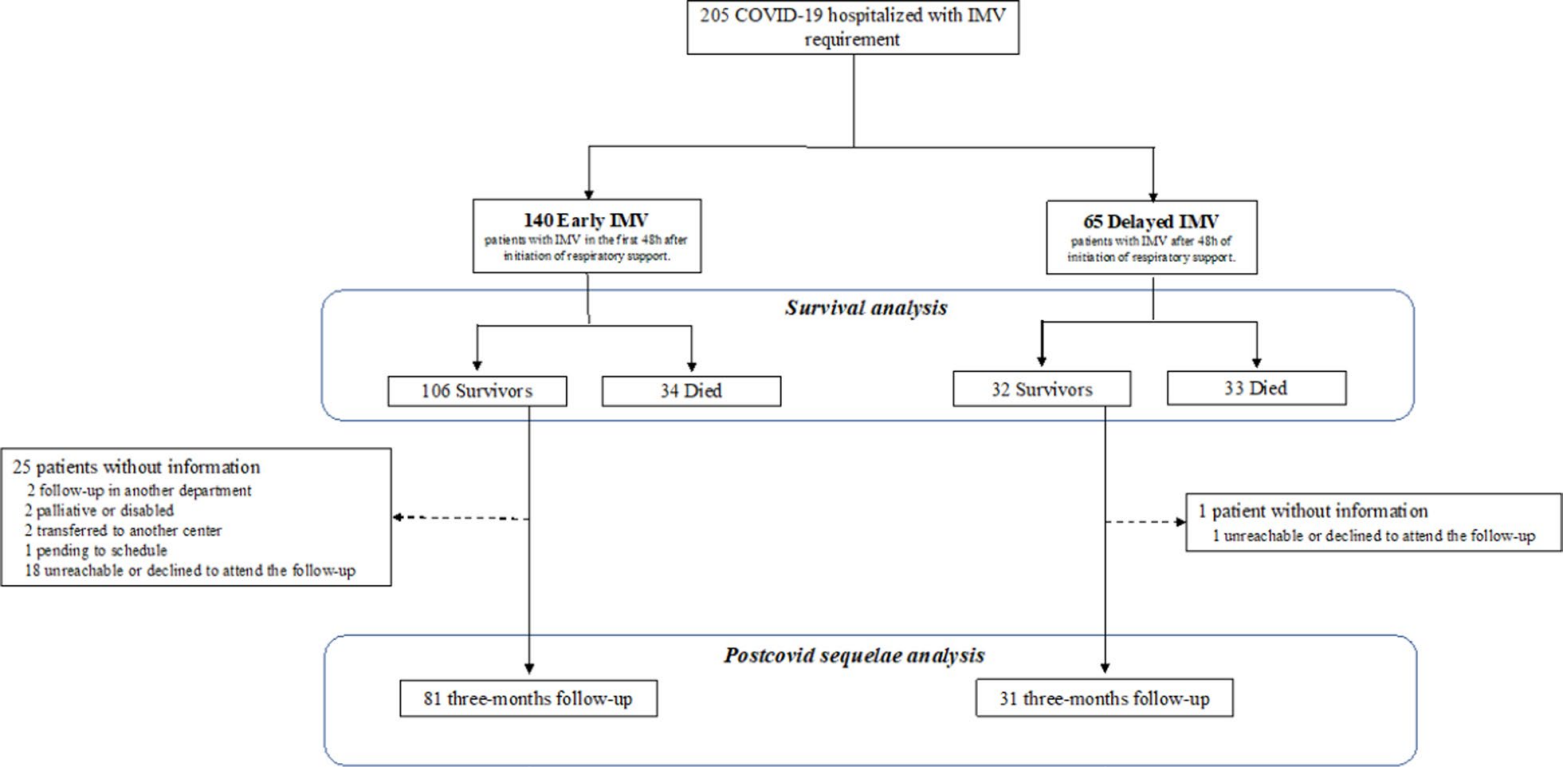
Flow chart of the study. *IMV* invasive mechanical ventilation

Figure 1 shows the flowchart of the study. Between March 2020 and February 2021, 205 patients required intubation during their ICU stay due to COVID-19, 140 had early IMV and 65 had delayed IMV. One hundred and six patients survived in the early IMV group, and 32 survived in the delayed group. The causes of death in each group are shown in Additional file 1: Table S1. Eighty-one and 31 of these patients, respectively, were followed and completed the pulmonary evaluation.

### Characteristics of the cohort

The final cohort of 205 patients was characterized as being middle aged (median [p_25_; p_75_] of 63.0 [56.0; 70.0] years old) men (74.1%). The most frequent comorbidities were hypertension (53.7%), obesity (52.9%) and diabetes mellitus (31.7%). The majority of patients presented acute respiratory distress syndrome (ARDS) at hospital admission (87.1%) with a P_a_O_2_/F_I_O_2_ ratio of 200 [128; 257]. Regarding the differences between study groups, a significantly greater number of patients belonging to the early group were diabetic, had a worse P_a_O_2_/F_I_O_2_ ratio at hospital admission, greater inflammatory biological variables (CPR and D-Dimer) and received different pharmacological treatments (Table 1).

**Table 1:** Patient characteristics at hospital admission

	Global	Early IMV	Delayed IMV	*p* value	*N*
	*n* = 205 Median [IQR] or *n* (%)	*n* = 140 Median [IQR] or *n* (%)	*n* = 65 Median [IQR] or *n* (%)		
*Sociodemographic data*					
Sex, woman	53 (25.9%)	34 (24.3%)	19 (29.2%)	0.561	205
Age, years	63.0 [56.0; 70.0]	63.0 [54.0; 71.0]	63.0 [59.0; 69.0]	0.380	205
Smoking history				0.061	183
Nonsmoker	99 (54.1%)	75 (59.5%)	24 (42.1%)		
Current	11 (6.01%)	8 (6.35%)	3 (5.26%)		
Former	73 (39.9%)	43 (34.1%)	30 (52.6%)		
*Comorbidities*					
Obesity	108 (52.9%)	78 (56.1%)	30 (46.2%)	0.239	204
Hypertension	110 (53.7%)	77 (55.0%)	33 (50.8%)	0.678	205
Diabetes mellitus	65 (31.7%)	52 (37.1%)	13 (20.0%)	**0.022**	205
Chronic heart disease	26 (12.7%)	22 (15.7%)	4 (6.15%)	0.091	205
Chronic renal disease	12 (5.85%)	7 (5.00%)	5 (7.69%)	0.525	205
COPD/bronchiectasis	18 (8.78%)	13 (9.29%)	5 (7.69%)	0.912	205
Asthma	12 (5.85%)	9 (6.43%)	3 (4.62%)	0.756	205
Immunologic disease	4 (1.95%)	2 (1.43%)	2 (3.08%)	0.593	205
*Hospital admission*					
Days from the firsts symptoms	7.00 [5.00; 9.00]	7.00 [5.00; 9.00]	6.00 [4.00; 8.00]	0.088	205
Laboratory data					
Urea nitrogen mg/dL	46.5 [32.0; 65.8]	48.0 [33.5; 61.2]	44.5 [31.8; 71.0]	0.881	200
Creatinine mg/dL	0.92 [0.76; 1.16]	0.92 [0.75; 1.13]	0.94 [0.78; 1.27]	0.469	204
C-reactive protein mg/dL	151 [87.3; 211]	155 [91.8; 239]	116 [68.5; 181]	**0.020**	195
White blood count × 109/L	7.27 [5.53; 10.2]	8.07 [6.04; 10.9]	6.12 [4.91; 8.11]	**0.002**	205
Hemoglobin g/dL	13.8 [12.6; 14.9]	13.6 [12.6; 14.9]	13.9 [12.4; 14.9]	0.671	205
Platelet count × 109/L	200 [154; 245]	206 [162; 244]	183 [142; 246]	0.094	204
Lymphocyte count × 109/L	0.75 [0.56; 1.02]	0.75 [0.55; 1.01]	0.72 [0.63; 1.02]	0.443	205
International normalized ratio (INR)	1.13 [1.07; 1.21]	1.13 [1.09; 1.24]	1.12 [1.05; 1.18]	0.058	195
D-dimer mg/L	345 [225; 617]	369 [246; 686]	283 [214; 429]	**0.028**	164
Arterial blood gas					
pH	7.45 [7.39; 7.48]	7.44 [7.36; 7.47]	7.46 [7.43; 7.49]	**0.002**	182
Partial pressure of oxygen (PaO_2_)	66.0 [53.0; 90.0]	68.0 [54.0; 94.0]	61.5 [52.8; 81.0]	0.098	181
Partial pressure of carbon dioxide (PaCO_2_)	34.0 [31.0; 40.0]	35.0 [31.0; 41.2]	32.5 [30.0; 36.0]	**0.003**	180
Oxygen saturation (O_2_ Sat)	94.0 [89.9; 97.2]	94.2 [89.9; 97.4]	93.7 [90.0; 96.8]	0.310	196
PaO_2_ to FiO_2_ ratio	200 [128; 257]	176 [115; 238]	233 [160; 283]	**0.004**	187
APACHE score at ICU admission	14.0 [11.0; 18.0]	16 [13; 21]	12 [10; 15]	**< 0.001**	147
*Pharmacological treatment*					
Hydroxychloroquine	68 (33.2%)	61 (43.6%)	7 (10.8%)	**< 0.001**	205
Corticosteroids	189 (92.2%)	124 (88.6%)	65 (100%)	**0.011**	205
Anticoagulant	192 (93.7%)	129 (92.1%)	63 (96.9%)	0.234	205
Antibiotics	191 (93.6%)	135 (96.4%)	56 (87.5%)	**0.027**	204
Lopinavir/ritonavir	68 (33.2%)	61 (43.6%)	7 (10.8%)	**< 0.001**	205
Remdesivir	27 (13.2%)	16 (11.5%)	11 (16.9%)	0.400	204
Tocilizumab	129 (62.9%)	77 (55.0%)	52 (80.0%)	**0.001**	205

Numbers in bold are statistically significant *p*-values

*COPD* chronic obstructive pulmonary disease, *IQR* interquartile range, *IMV* invasive mechanical ventilation, *ICU* intensive care unit

### Time from the initiation of the first respiratory support to IMV

In general, patients quickly required their first respiratory support (with NIMV, HFNC or IMV) after a median of 0 [0; 1] days of hospital admission. At this point, patients presented poor oxygenation with a median (SD) P_a_O_2_/F_I_O_2_ ratio of 106.5 [80.5; 143.0]. Initiation of the first ventilatory support was carried out in the general ward for 50.8% of the delayed group, but was conducted in the ICU for 77.0% of the early IMV group. The initial support strategy was IMV (13.6%), HFNC (45.9%) and NIVM (40.5%) for the whole cohort. After stratification by group, the initial support strategy was HFNC (41.4% vs. 54.4%) and NIVM (38.6% vs. 44.6%) in the early IMV and delayed IMV groups, respectively. Patients who started the first ventilatory support in the general ward were generally admitted to the ICU in the next 24 h, with a median time of 1 [1; 2] days. On the day of NIMV initiation, patients in the early group showed worse ROX indexes than the delay group, with a median of 3.55 [2.2; 5.4] vs 5.70 [4.4; 7.3], respectively (Table 2). Patients in the early group were intubated 48 h after the start of the first ventilatory support, while a median of 4.00 [3.00; 6.00] days elapsed in the delayed group. Nevertheless, no differences in the time from the first ventilation to IMV were observed between those who started with HFNC and NIMV (data not shown). On the day of IMV, patients showed worsening oxygenation with a median P_a_O_2_/F_I_O_2_ ratio of 74.0 [61.0; 99.0], without differences between study groups (Table 2). Additionally, patients in the early group had worse punctuation in the APACHE score (median of 16 [13; 21] vs 12 [10:15]) (Table 1). No differences were observed in respiratory mechanics (tidal volume, end-inspiratory plateau and peak inspiratory pressures, PEEP, driving pressure and Crs) on the day of intubation between both groups (Table 2).

**Table 2:** Respiratory support strategies

	Early IMV	Delayed IMV	*p* value	*N*
	*n* = 140 Median [IQR] or *n* (%)	*n* = 65 Median [IQR] or *n* (%)		
*Initial ventilatory support*				
Ventilation start site			**< 0.001**	205
General ward	31 (22.1%)	33 (50.8%)		
ICU	109 (77.9%)	32 (49.2%)		
Time from hospital admission to initial ventilatory support (days)	0.00 [0.00; 1.00]	0.00 [0.00; 1.00]	**0.019**	205
PaO_2_ to FiO_2_ ratio	98.8 [70.0; 132]	129 [100; 150]	**< 0.001**	183
Respiratory rate	30.0 [24.0; 36.0]	24.0 [20.0; 27.8]	**< 0.001**	171
Respiratory support management prior to intubation			**< 0.001**	205
None	28 (20.0%)	0 (0.00%)		
High-flow nasal cannula	39 (27.9%)	2 (3.08%)		
CPAP/BIPAP	29 (20.7%)	3 (4.62%)		
Both therapies	44 (31.4%)	60 (92.3%)		
ROX index	3.55 [2.26; 5.43]	5.70 [4.45; 7.33]	**< 0.001**	140
*IMV*				
Time from hospitalization to IMV (days)	1.00 [0.00; 2.00]	5.00 [4.00; 7.00]	**< 0.001**	205
Time from initial ventilatory support to IMV (days)	1.00 [0.00; 1.00]	4.00 [3.00; 6.00]	**< 0.001**	205
PaO_2_ to FiO_2_ ratio	74.0 [61.2; 99.0]	74.0 [60.0; 91.0]	0.560	195
Respiratory rate	29.0 [22.0; 35.0]	25.0 [22.0; 30.0]	**0.019**	148
Duration (days)	13.0 [7.00; 24.0]	17.0 [7.75; 30.0]	0.431	202
Prone positioning	106 (75.7%)	53 (81.5%)	0.453	205
Ventilatory setting and pulmonary mechanics				
Tidal volume/PBW (mL/Kg)	5.32 [4.66; 6.09]	5.42 [4.87; 6.05]	0.665	158
PEEP, cmH_2_O	14 [12; 15]	13 [12; 14]	0.421	168
Peak inspiratory pressure, cmH_2_O	34 [30; 37]	32 [29; 36]	0.233	163
End-inspiratory plateau pressure, cmH_2_O	25 [22; 29]	24.5 [19; 28]	0.261	145
Driving pressure, cmH_2_O^a^	12 [8; 14]	10.5 [6.75; 14]	0.358	145
Compliance, mL/cmH_2_O^b^	39.2 [32; 57]	45.5 [35; 74]	0.232	142

Numbers in bold are statistically significant *p*-values

*ICU* intensive care unit, *CPAP* continuous positive airway pressure, *BIPAP* bilevel positive airway pressure, *IQR* interquartile range, *IMV* invasive mechanical ventilation, *ROX* respiratory rate-OXygenation, *PEEP* positive end-expiratory pressure, *PBW* predicted body weight

^a^Defined as plateau pressure-PEEP

^b^Defined as tidal volume/(Plateau pressure − PEEP)

### In-hospital mortality according to the IMV delay

**Fig. 2:**
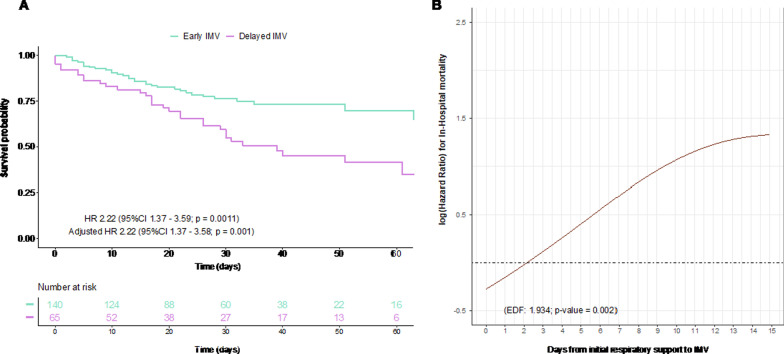
In-hospital mortality according to the IMV delay. **A** Kaplan–Meier curves for in-hospital overall survival. **B** Additive Cox proportional hazard model with a cubic spline basis to evaluate the association between the time from initial ventilatory support to IMV and in-hospital mortality. *EDF* effective degrees of freedom, *IMV* invasive mechanical ventilation

**Fig. 3:**
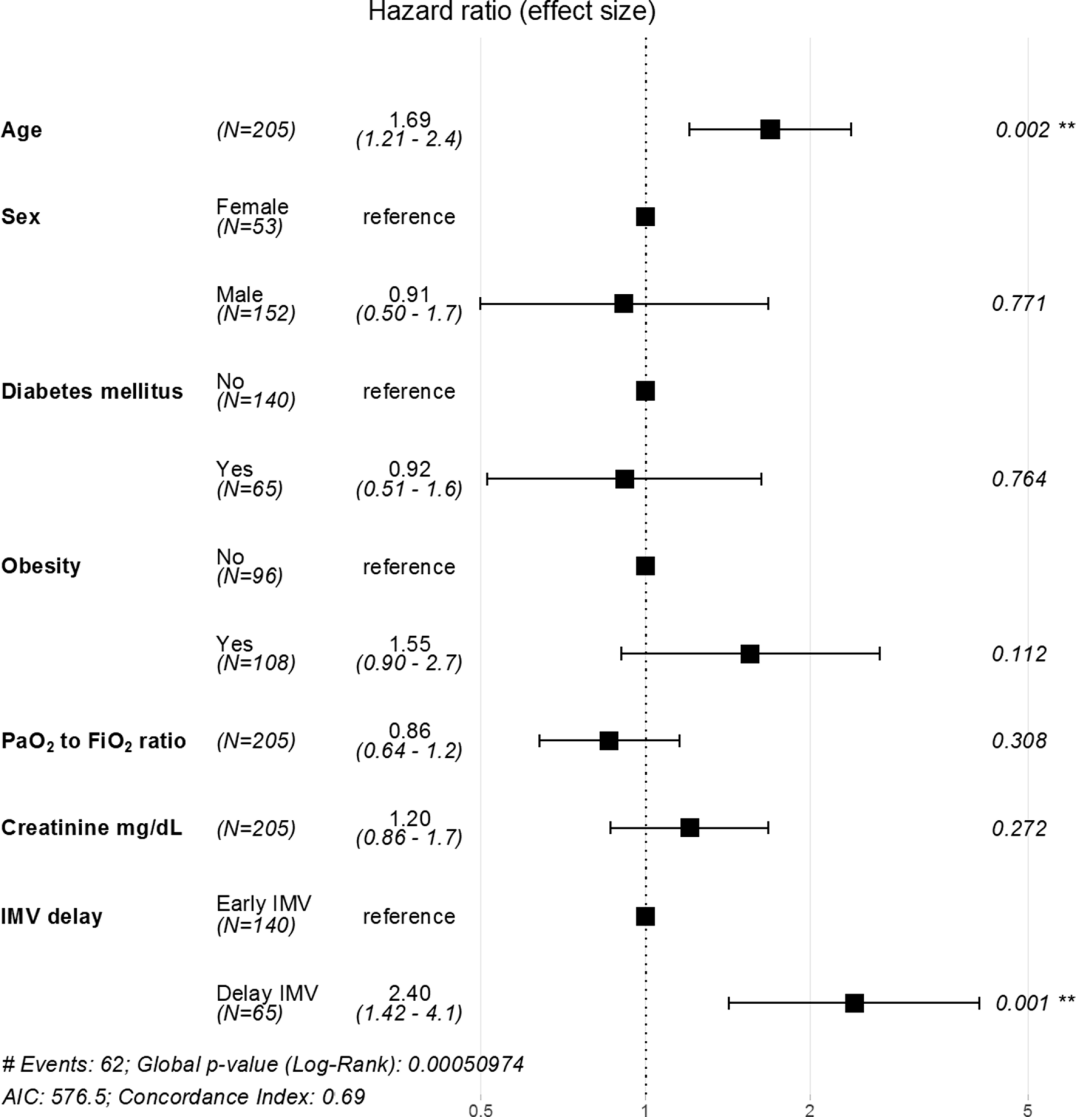
Multivariate Cox model with predictors of mortality risk. *IMV* invasive mechanical ventilation

The ICU mortality of the study groups was 24.3% and 50.8% in the early IMV and delayed groups, respectively (odds ratio of 3.19 [95% CI 1.65–6.26]). Furthermore, the survival analysis showed a significant increase in the risk of mortality in the delayed group, with a hazard ratio (HR) of 2.2 (95% CI 1.37–3.58; p = 0.001) (Fig. 2A). Similarly, an HR of 2.45 (95% CI 1.29–4.65) was obtained from the doubly robust adjustment method including the propensity score and confounding factors as covariates in the model. There were no differences in this observation between the different epidemic waves (from March to May 2020 vs June 2020 to February 2021) (Additional file 1: Table S2). Furthermore, an additive Cox proportional hazard model with time to IMV as a continuous predictor showed a nonlinear dose–response association with the risk of in-hospital mortality (Fig. 2B). The multivariate mortality model showed a significant effect of age, P_a_O_2_/F_I_O_2_ ratio at the initiation of IMV, creatinine level at hospital admission, and delay of IMV (Fig. 3). The hospital stay in the delay group was longer, with a median (IQR) of 39.5 [23.0; 59.0] days compared with 32 [21; 45] days in the early intubation group (*p* value = 0.105).

Additionally, we performed a competing risk analysis because in-hospital mortality risk can be overestimated when considering discharge as censored information (Additional file 1: Figure S1). These results were similar to those estimated in the survival analysis using the Cox model, showing an increased risk of in-hospital mortality with an adjusted subdistribution HR of 2.59 (95% CI 1.34–5).

### Sequelae of survivors according to the IMV delay

The pulmonary sequelae were evaluated after a median (p_25_; p_75_) of 103 (91; 123) days from hospital discharge. No difference in follow-up time was observed between study groups. The patients included in this analysis showed similar sociodemographic and clinical characteristics at hospital admission (Additional file 1: Table S3) to the patients who did not attend the follow-up visit. Regarding the differences in surviving patients between study groups, differences in pharmacological treatment and laboratory data on hospital admission were observed (Additional file 1: Table S4).

In general, patients showed a high degree of respiratory sequelae and lung damage (Table 3). Regarding functional sequelae, patients in the delayed group showed a worse DLCO than those in the early intubation group, with a mean difference of − 10.77 (95% CI − 18.40 to − 3.15). The delayed group showed greater changes in the lung on the CT scan of the thorax with a greater number of affected lobes (mean difference of 1.51 [95% CI 0.89–2.13]; *p* value < 0.001) and a greater TSS (mean difference of 4.35 [95% CI 2.41–6.27]; *p* value < 0.001). Oxygen saturation at the beginning of the 6MWT was lower in the delayed group (97% vs. 96%; *p* = 0.007).

**Table 3:** Sequelae of survivors according to the IMV delay

	ALL	Early IMV	Delayed IMV	*p* value	*N*
	*n* = 111 Median [IQR] or *n* (%)	*n* = 81 Median [IQR] or *n* (%)	*n* = 30 Median [IQR] or *n* (%)		
*Pulmonary function*					
FVC, %	79.6 [65.9; 88.3]	79.3 [67.2; 88.0]	80.1 [63.0; 89.8]	0.849	109
FEV1, %	86.1 [73.2; 96.1]	85.8 [74.2; 95.0]	87.8 [70.6; 100]	0.879	108
FEV1 to FVC ratio	0.83 [0.81; 0.86]	0.83 [0.80; 0.86]	0.84 [0.82; 0.87]	0.156	103
FEV1 to FVC ratio > 0.7	102 (99.0%)	77 (100%)	25 (96.2%)	0.252	103
TLC, % mean (sd)	81.1 [71.1; 88.0]	81.8 [66.2; 87.7]	78.4 [73.1; 88.1]	0.952	99
TLC, %				0.553	99
< 50%	5 (5.05%)	3 (4.11%)	2 (7.69%)		
≤ 50–80%	53 (53.5%)	41 (56.2%)	12 (46.2%)		
≥ 80%	41 (41.4%)	29 (39.7%)	12 (46.2%)		
RV %	81.0 [66.0; 101]	85.0 [65.9; 102]	74.5 [69.5; 94.9]	0.484	99
DLCO, mL/min/mm Hg mean (sd)	65.0 [55.0; 74.6]	68.4 [57.3; 76.7]	60.0 [51.5; 64.5]	**0.010**	107
DLCO, mL/min/mm Hg				0.246	107
< 60%	36 (33.6%)	23 (29.1%)	13 (46.4%)		
≤ 60–80%	53 (49.5%)	41 (51.9%)	12 (42.9%)		
≥ 80%	18 (16.8%)	15 (19.0%)	3 (10.7%)		
*6MWT*					
Distance, m	395 [320; 440]	395 [320; 430]	390 [325; 460]	0.825	104
Oxygen saturation, %					
Initial	97.0 [96.0; 97.0]	97.0 [96.0; 98.0]	96.0 [96.0; 97.0]	**0.005**	104
Final	96.0 [94.0; 96.0]	96.0 [94.0; 96.0]	95.0 [93.0; 96.0]	0.080	104
Average	96.0 [94.0; 96.0]	96.0 [94.0; 96.0]	95.0 [94.0; 97.0]	0.598	104
Minimal	94.0 [92.0; 96.0]	94.0 [92.0; 96.0]	94.0 [92.0; 95.0]	0.281	104
*Chest CT scan findings*					
Density					
Ground glass	50 (46.3%)	36 (45.6%)	14 (48.3%)	0.974	108
Mixed ground glass	50 (46.3%)	29 (36.7%)	21 (72.4%)	**0.002**	108
Consolidation	22 (20.4%)	14 (17.7%)	8 (27.6%)	0.391	108
Internal structures					
Interlobular septal thickening	93 (86.1%)	66 (83.5%)	27 (93.1%)	0.346	108
Bronchiectasis	89 (82.4%)	64 (81.0%)	25 (86.2%)	0.731	108
Atelectasis	31 (28.7%)	25 (31.6%)	6 (20.7%)	0.381	108
Solid nodule	44 (40.7%)	29 (36.7%)	15 (51.7%)	0.235	108
Nonsolid nodule	3 (2.78%)	2 (2.53%)	1 (3.45%)	1.000	108
Lesions				**0.004**	108
Reticular	45 (41.7%)	39 (49.4%)	6 (20.7%)		
Fibrotic	43 (39.8%)	24 (30.4%)	19 (65.5%)		
None	20 (18.5%)	16 (20.3%)	4 (13.8%)		
Number of lobes affected	5.00 [2.00; 5.00]	4.00 [2.00; 5.00]	5.00 [5.00; 5.00]	**< 0.001**	108
Total severity score	7.00 [3.00; 10.0]	6.00 [2.00; 9.50]	10.0 [7.00; 12.0]	**< 0.001**	108

Numbers in bold are statistically significant *p*-values

*FVC* forced expiratory volume, *FEV1* forced expiratory volume during the first second of the forced breath, *RV* residual volume, *DLCO* diffusing capacity of lung for carbon monoxide, *6MWT* Six Minute Walk Test, *IQR* interquartile range, *IMV* invasive mechanical ventilation

The selection of important characteristics at hospital admission based on the random forest model to predict functional and structural sequelae was carried out. The final model included the delay in intubation and IMV days as important variables to predict TSS at the follow-up visit (Additional file 1: Figure S2A). Similarly, hospital stay, smoking and delay in intubation were selected to predict DLCO at follow-up (Additional file 1: Figure S2B).

## Discussion

In this prospective and well-characterized cohort of intubated patients due to CARDS, the delay in intubation (> 48 h from the first respiratory support) had important implications for in-hospital mortality and pulmonary sequelae during the follow-up of survivors. Patients with delayed IMV exhibited a doubled risk of death with a dose–response relationship between an increased risk and a longer delay. In the multivariate mortality model, factors such as age, creatinine levels at hospital admission, P_a_O_2_/F_I_O_2_ ratio and delay in intubation exerted a significant effect on mortality. Importantly, patients who survived and belonged to the delayed group presented the most severe pulmonary sequelae with the worst DLCO and greater changes on chest CT, with a greater number of affected lobes and greater TSS.

In a multi-intensive care unit prospective cohort of 457 patients with ARDS [31], patients who were intubated within 3 days had a higher mortality rate than those intubated early. Importantly, this difference persisted for 2 years of follow-up. The authors chose the cutoff of 3 days, but the majority of the late intubation group underwent intubation on Day 2, precisely when mortality was increasing dramatically.

Regarding CARDS, the evidence also indicates that increasing the time from admission to intubation is associated with higher mortality rates in patients requiring mechanical ventilation. Hyman et al*.* [32] evaluated the association between the time from hospital admission to IMV and mortality due to CARDS in five hospitals in New York City. They showed a significant association between the timing of intubation and improvement in survival. Specifically, they found a 3% increase in mortality for each day of delay in intubation following hospital admission. In another study, the difference at intubation timing was 18% in < 48 h versus 43% in > 48 h (*p* < 0.01) [33]. These results are consistent with our study and highlight the importance of not delaying intubation once patients develop CARDS. Similarly, an important study using propensity score analysis to assess the risk and benefits of the different respiratory support strategies employed in the ICU and their timing has recently been published [24]. The authors found that patients initially treated with NIMV who subsequently required intubation had a higher ICU mortality rate (37%) than patients treated with the other strategies (standard oxygen therapy: 21%, HFNC: 31%) compared to the early group (intubated within the first 24 h of ICU admission). Consequently, their conclusion of the optimal initial ventilatory strategy is to try a close monitored trial period of HFNC but prioritize rapid IMV in people at high risk of failure. However, the authors considered the baseline time of the study as ICU admission, and thus respiratory support management conducted in the general ward before ICU admission was not considered. Because the use of NIMV or HFNC outside the ICU has been widely accepted during the COVID-19 pandemic [13], we proposed a different approach (the first 48 h from the first respiratory support), which might be more appropriate and fits with real clinical practice.

Some plausible explanations for the increased mortality due to prolonged use of noninvasive ventilatory support and delayed intubation in patients who ultimately fail and require IMV are proposed. Patients with ARDS and CARDS initiate a vicious cycle through spontaneous vigorous inspiratory efforts associated with high transpulmonary pressures that lead to excess stress and increased pulmonary inflammation [34, 35], contributing to the worsening of lung damage (patient self-induced lung injury or P-SILI) [10]. Classically, lung-protective ventilation through sedation and IMV has been applied to minimize the progression of lung injury to a form of P-SILI [35].

CARDS has been proposed as an “atypical ARDS” due to many factors but importantly because of the dissociation of relatively well-preserved lung mechanics and the severity of hypoxemia [10, 11]. In fact, a time-related disease spectrum within two primary “phenotypes” has been postulated [36]. Initially, COVID-19 pneumonia can be categorized in type L, grouping together patients with lungs showing low elastance, low ventilation-to-perfusion (VA/Q) ratio, low lung weight and low lung recruitability [36]. After this stage, patients may improve or evolve to type H with characteristics totally opposed to the latter. These patients have lungs with high elastance and lung weight, high lung recruitability and a high right-to-left shunt [36].

The transition from type L to type H may be due to both the evolution of the severity of COVID-19 pneumonia and the injury attributable to the aforementioned P-SILI, secondary to high negative intrathoracic pressure and increased tidal volume during spontaneous breathing [35, 36]. Early intubation, effective sedation and/or paralysis may interrupt this cycle. Therefore, respiratory support treatment should differ according to the different phenotypes and stages of the disease. Type L patients should benefit from noninvasive options with HFNC or NIV. However, surrogate measures for the work of breathing or clinical detection of excessive inspiratory effort should be evaluated to avoid delaying IMV. Overall, the respiratory support strategy could very plausibly contribute to and may play a key role in the presence and severity of pulmonary sequelae in the follow-up of these critically ill patients with COVID-19.

Our study has several limitations. First, we analyzed a small cohort from a single city, and a larger sample size from different hospitals would be ideal for this type of study. Second, this study only included patients who underwent IMV, and it does not provide insights into those who responded well on NIMV and never required intubation. Future studies are needed to determine whether the use of NIMV support reduce the need for intubation in patients with CARDS and its effect on mortality. Third, even though a PS was performed to try to make the two groups comparable, it is possible that some small differences between baseline characteristics and disease progression have a subtle effect on the results.

## Conclusions

In conclusion, intubation timing exerts important effects on both in-hospital mortality and pulmonary sequelae during the follow-up of survivors. These findings have several clinical implications and provide a basis for future studies to improve the respiratory management of patients with CARDS.

## Supplementary Information

**Additional file 1**. Description of the population and additional information on variables and results.

## Data Availability

Not applicable.
