# Supplementary material for: Impact of time to intubation on mortality and pulmonary sequelae in critically ill patients with COVID-19: a prospective cohort study
Source: Crit Care. 2022 Jan 10;26:18. doi: 10.1186/s13054-021-03882-1 (PMC8744383; doi:10.1186/s13054-021-03882-1)
Supplement: Supplementary file 1 — Additional file 1. Description of the population and additional information on variables and results. [file 13054_2021_3882_MOESM1_ESM.docx]

***Online Supplement***

**IMPACT OF TIME TO INTUBATION ON MORTALITY AND PULMONARY SEQUELAE IN CRITICALL ILL COVID-19 PATIENTS: a prospective cohort.**

Jessica González^1,2,3,4^, Iván D. Benítez^2,3,4^, David de Gonzalo-Calvo^2,3,4^, Gerard Torres^1,2,3,4^, Jordi de Batlle^2,3,4^, Silvia Gómez^1,2,3,4^, Anna Moncusí-Moix^2,3,4^, Paola Carmona^1,2,3,4^ Sally Santisteve^1,2,3,4^, Aida Monge^1,2,3,4^, Clara Gort-Paniello^2,3,4^, María Zuil^1,2,3,4^, Ramón Cabo-Gambín^1,2,3,4^, Carlos Manzano Senra^1,2,3,4^, José Javier Vengoechea Aragoncillo^1,2,3,4^, Rafaela Vaca^1,2^, Olga Minguez^1,2^, María Aguilar^1,2^, Ricard Ferrer^4,5^, Adrián Ceccato^4^, Laia Fernández^4,6^, Ana Motos,^4,6^ Jordi Riera,^4,5^ Rosario Menéndez,^4,7^ Darío Garcia-Gasulla,^8^ Oscar Peñuelas,^4,9^ Jesús F. Bermejo-Martin,^4,10^ Gonzalo Labarca,^11^ Jesús Caballero^12^, Carme Barberà^13^, Antoni Torres,^4,6^ Ferran Barbé,^1,2,3,4^ *on behalf of the CIBERESUCICOVID Project (COV20/00110, ISCIII)*

**Figure S1. Competing Risk analysis.**





**Table S1. Causes of death according to the different groups**

**Table S2. IOT timing effect between the different epidemic waves (from March to May 2020 vs June 2020 to February 2021)**

**Table S3. Sociodemographic and clinical characteristics at hospital admission between patients who were lost to follow-up and those who attended the consultation.**

**Table S4. Sociodemographic and clinical characteristics at hospital admission of surviving patients stratified according to the study group.**

**Figure S2. Selection process for important variables predicting functional and structural sequelae based on the random forest model.** (A) Importance of each variable to predict DLCO at the follow-up visit (right panel) and selection of the best combination of variables that provides the least error rate to predict DLCO at the follow-up visit (left panel). (B) Importance of each variable to predict TSS at the follow-up visit (right panel) and selection of the best combination of variables that provides the least error rate to predict TSS at the follow-up visit (left panel).

OOB = Out-of-bag. DLCO = diffusing capacity of the lung for carbon monoxide. TSS = total severity score. IMV=invasive mechanical ventilation. HIV=human immunodeficiency virus.
